# p63 expression in human tumors and normal tissues: a tissue microarray study on 10,200 tumors

**DOI:** 10.1186/s40364-021-00260-5

**Published:** 2021-01-25

**Authors:** Stefan Steurer, Claudia Riemann, Franziska Büscheck, Andreas M. Luebke, Martina Kluth, Claudia Hube-Magg, Andrea Hinsch, Doris Höflmayer, Sören Weidemann, Christoph Fraune, Katharina Möller, Anne Menz, Margit Fisch, Michael Rink, Christian Bernreuther, Patrick Lebok, Till S. Clauditz, Guido Sauter, Ria Uhlig, Waldemar Wilczak, David Dum, Ronald Simon, Sarah Minner, Eike Burandt, Rainer Krech, Till Krech, Andreas H. Marx

**Affiliations:** 1 Institute of Pathology, University Medical Center Hamburg-Eppendorf, Martinistr. 52, 20246 Hamburg, Germany; 2 Department of Urology, University Medical Center Hamburg-Eppendorf, Hamburg, Germany; 3 Institute of Pathology, Clinical Center Osnabrueck, Osnabrueck, Germany; 4 Department of Pathology, Academic Hospital Fuerth, Fuerth, Germany

## Abstract

**Background:**

Tumor protein 63 (p63) is a transcription factor of the p53 gene family involved in differentiation of several tissues including squamous epithelium. p63 immunohistochemistry is broadly used for tumor classification but published data on its expression in cancer is conflicting.

**Methods:**

To comprehensively catalogue p63 expression, tissue microarrays (TMAs) containing 12,620 tissue samples from 115 tumor entities and 76 normal tissue types were analyzed.

**Results:**

p63 expression was seen in various normal tissues including squamous epithelium and urothelium. At least occasional weak p63 positivity could be detected in 61 (53%) of 115 different tumor types. The frequencies of p63 positivity was highest in squamous cell carcinomas irrespective of their origin (96–100%), thymic tumors (100%), urothelial carcinomas (81–100%), basal type tumors such as basal cell carcinomas (100%), and various salivary gland neoplasias (81–100%). As a rule, p63 was mostly expressed in cancers derived from p63 positive normal tissues and mostly not detectable in tumors derived from p63 negative cancers. However, exceptions from this rule occurred. A positive p63 immunostaining in cancers derived from p63 negative tissues was unrelated to aggressive phenotype in 422 pancreatic cancers, 160 endometrium cancers and 374 ovarian cancers and might be caused by aberrant squamous differentiation or represent stem cell properties. In 355 gastric cancers, aberrant p63 expression occurred in 4% and was linked to lymph node metastasis (*p* = 0.0208). Loss of p63 in urothelial carcinomas - derived from p63 positive urothelium - was significantly linked to advanced stage, high grade (*p* < 0.0001 each) and poor survival (*p* < 0.0001) and might reflect clinically relevant tumor dedifferentiation.

**Conclusion:**

The high prevalence of p63 expression in specific tumor types makes p63 immunohistochemistry a suitable diagnostic tool. Loss of p63 expression might constitute a feature of aggressive cancers.

**Supplementary Information:**

The online version contains supplementary material available at 10.1186/s40364-021-00260-5.

## Introduction

Tumor protein 63 (p63) is a transcription factor of the p53 gene family encoded by the TP63 gene located at chromosome 3q28. p63 regulates the activity of a multitude of genes involved in growth and development of the ectoderm and derived structures and tissues, such as basal layer keratins and cell cycle control genes [1]. Accordingly, p63 expression is found in basal cell layers of various organs, squamous epithelial cells of many organs and urothelium [1–4]. p63 (syn. TAp63) is closely related to p40 (syn. ΔNp63) as both proteins represent isoforms of the p63 gene with distinct molecular functions [3]. While “full length” p63 (TAp63) activates p53 target genes such as p21 or BAX [4], the shorter transcript p40 (ΔNp63) inhibits activation of p53 and “full length” p63 [4–6].

In diagnostic pathology, the consistently high expression of p63 in specific cell and tissue types is exploited for diagnostic purposes. For example, p63 immunohistochemistry (IHC) is commonly used to mark cell types with critical impact on cancer diagnosis such as basal cells in prostatic and breast glands. Together with other antibodies, p63 is also routinely used for tumor type determination, for example distinguishing squamous cell carcinoma from adenocarcinoma in lung biopsies, or urothelial carcinomas from renal cell carcinoma in tumors arising in the kidney as well as determining the tumor origin of metastases from unknown primary tumors. Since the first description of p63 antibodies, more than 2000 studies have evaluated p63 expression by IHC in various tumors leading to quite discrepant p63 positivity rates in a number of tumor entities [2, 7–77]. For example, the fraction of p63 positive cases ranged from 0 to 77% in small cell lung cancer [19, 78], from 50 to 100% in squamous cell lung cancer [22, 52], 0 to 84% in Merkel cell carcinoma [30, 56], 0 to 82% in papillary thyroid carcinoma [36, 74], 1.4 to 100% in colorectal adenocarcinoma [9, 45], 0 to 100% in urothelial carcinoma [41, 76], 0 to 100% in mucinous ovarian carcinoma [7, 79], and from 0 to 25% in endometroid ovarian carcinoma [7, 79]. These conflicting data are likely to be caused by the use of different antibodies, immunostaining protocols, and criteria to determine p63 positivity in these studies.

To better understand the relative importance of p63 expression in different tumor types and normal tissues, a comprehensive study analyzing a large number of neoplastic and non-neoplastic tissues under highly standardized conditions is needed. We thus analyzed p63 expression in more than 12,000 tumor tissue samples from 115 different tumor types and subtypes as well as 76 non-neoplastic tissue types by IHC in a tissue microarray (TMA) format.

## Materials and methods

### Tissue microarrays (TMAs)

Our normal tissue TMA was composed of 8 samples from 8 different donors for each of 76 different normal tissue types (608 samples on one slide). The cancer TMAs contained a total of 12,620 primary tumors from 115 tumor types and subtypes. Detailed histopathological data on grade, pT and pN status were available from 1708 cancers (stomach, pancreas, ovarian, endometrium, urinary bladder). Clinical follow up data were only available from 254 patients who had undergone cystectomy for muscle invasive (pT ≥ 2) urinary bladder cancer. In these patients the median follow-up time was 14 (range 1–77) months. Clinical follow up data from patients with carcinomas of the stomach, pancreas, ovarian, and endometrium are not available. The composition of both normal and cancer TMAs is described in detail in the results section. All samples come from the archives of the Institutes of Pathology of the University Hospital of Hamburg, Germany, Clinical Center Osnabrueck, Germany, and the Academic Hospital Fuerth, Germany. No other information beyond the histological tumor type was available for these samples. Tissues were fixed in 4% buffered formalin and then embedded in paraffin. For TMA manufacturing a tumor containing donor block (at least 70% tumor cells in a sufficiently large area) of each patient was selected. Per tumor block/patient one TMA tissue spot (diameter: 0.6 mm) was transferred to an empty recipient TMA block. The use of remnants of archived diagnostic tissues for manufacturing of TMAs and their analysis for research purposes as well as patient data analysis has been approved by local laws (HmbKHG, §12) and by the local ethics committee (Ethics commission Hamburg, WF-049/09). All work has been carried out in compliance with the Helsinki Declaration.

### Immunohistochemistry

Freshly cut TMA sections were immunostained on one day and in one experiment. Slides were deparaffinized and exposed to heat-induced antigen retrieval for 5 min in an autoclave at 121 °C in pH 7.8 buffer. Primary antibody specific for p63 (clone 7B4, dilution 1:100) was applied at 37 °C for 60 min. Bound antibody was then visualized using the EnVision Kit (Agilent, CA, USA) according to the manufacturer’s directions. For tumor tissues, the percentage of nuclear positive neoplastic cells was estimated, and the staining intensity was semiquantitatively recorded (0, 1+, 2+, 3+). For statistical analyses, the staining results were categorized into four groups. Tumors without any staining were considered negative. Tumors with 1+ staining intensity in ≤70% of cells and 2+ intensity in ≤30% of cells were considered weakly positive. Tumors with 1+ staining intensity in > 70% of cells, 2+ intensity in 31–70%, or 3+ intensity in ≤30% were considered moderately positive. Tumors with 2+ intensity in > 70% or 3+ intensity in > 30% of cells werde considered strongly positive.

### Statistics

Statistical calculations were performed with JMP 14 software (SAS Institute Inc., NC, USA). Contingency tables and the chi^2^-test were performed to search for associations between p63 and tumor phenotype. Survival curves were calculated according to Kaplan-Meier. The Log-Rank test was applied to detect significant differences between groups. A significant difference was assumed for a *p*-value of ≤0.05.

## Results

### Technical issues

A total of 10,200 (80.8%) of 12,620 tumor samples were interpretable in our TMA analysis. Non-interpretable samples either lacked unequivocal tumor cells or were lost from the TMA during the technical procedures. A statistical bias, which could potentially result from exclusion of non-interpretable samples, is highly unlikely in our study as non-interpretable samples were evenly distributed across all pathological diagnoses (Supplementary Table 1). On our normal tissue TMA, a sufficient number of samples was always interpretable per tissue type to determine the normal tissue p63 expression.

### p63 in normal tissues

**Fig. 1:**
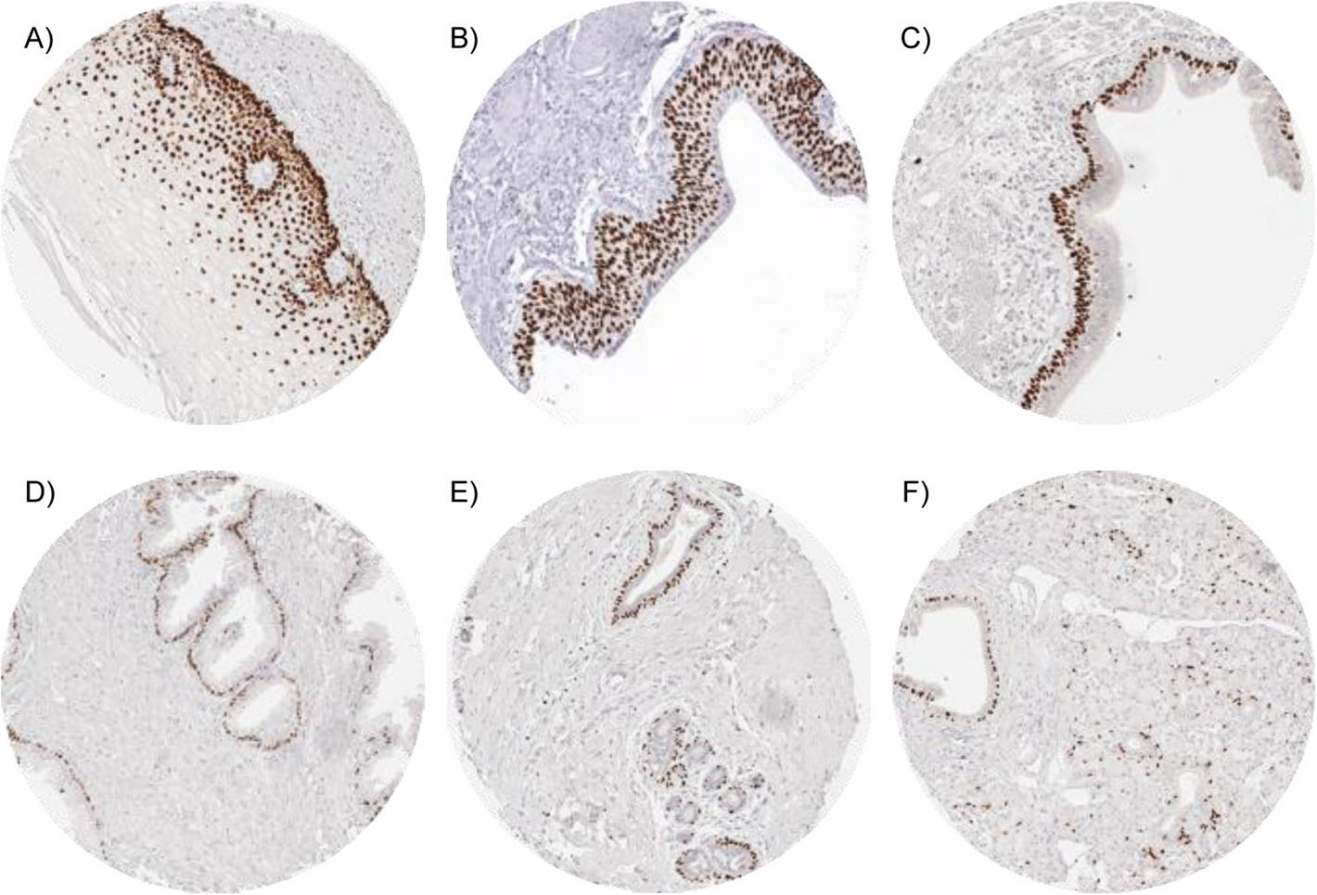
p63 expression in normal tissues. Strong p63 immunostaining is seen in squamous epithelium of the esophagus (**a**) urothelium of the urinary bladder (**b**), basal cells of respiratory epithelium (**c**) and of the prostate (**d**) and myoepithelial cells of the breast (**e**) and of the salivary glands (**f**)

A strong nuclear p63 immunostaining was seen in squamous epithelium irrespective of its origin, peripheric germinative cells of sebaceous glands, urothelium, thymic epithelial cells, myoepithelial cells in breast, parotid, submandibulary and sublingual glands, basal cells in prostate, seminal vesicle, and respiratory epithelium, cytotrophoblast of first trimenon and mature placenta. The staining intensity was slightly decreasing from basal cells to the surface cell layer in squamous epithelia and in urothelium. A mild staining was seen in some lymphocytes and in high endothelial venules in lymph nodes. Representative images are given in Fig. 1. p63 staining was absent in aorta/intima, aorta/media, heart (left ventricle), striated muscle, skeletal muscle, skeletal muscle/tongue, uterus/myometrium, muscular wall of the GI-tract, renal pelvis and bladder, corpus spongiosum of the penis, ovarian stroma, fat, red and white pulp of the spleen, antrum and corpus of the stomach, mucosa and lamina propria of the stomach duodenum, ileum, appendix, colon descendens, rectum, and gallbladder, epithelium of the gallbladder, liver, pancreas, bone marrow, Brunner gland of the duodenum, cortex and medulla of the kidney, epididymis, testis, glands of the bronchus, endocervix, proliferative and secretory endometrium, mucosa of the fallopian tube, corpus luteum and follicular cyst of the ovary, adrenal gland, parathyroid, thyroid gland, stratum molecular and neuronorum of the cerebellum, grey and white cerebrum, and posterior and anterior lobe of the pituitary gland.

### p63 in neoplastic tissues

**Fig. 2:**
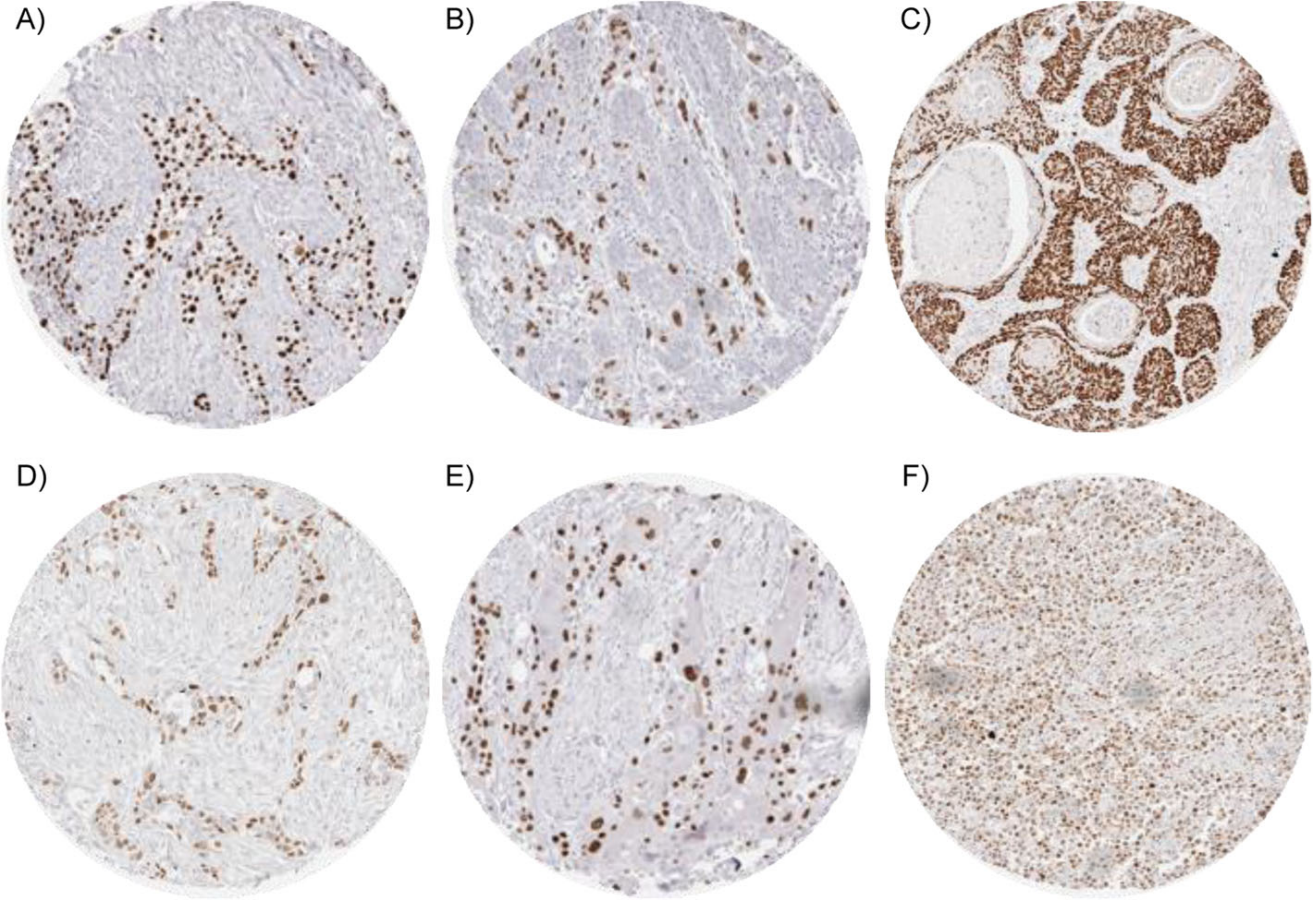
p63 expression in cancerous tissues. Strong p63 immunostaining is seen in invasive urothelial carcinoma (**a**) gastric cancer, diffuse type (**b**), anal squamous cell carcinoma (**c**), gastric cancer, intestinal type (**d**), and adenocarcinoma of the pancreas with focal squamous differentiation (**e**). Moderate intensity p63 staining is seen in a diffuse large B-cell lymphoma (**f**)

**Fig. 3:**
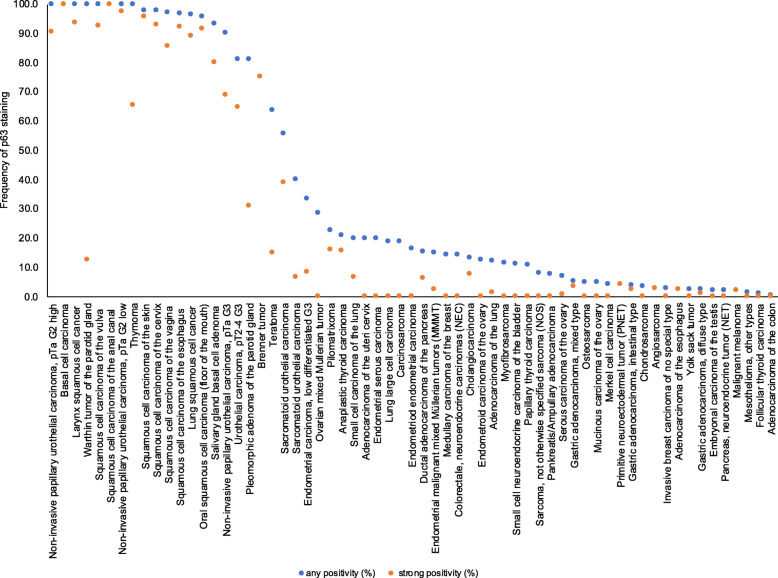
Ranking order of p63 immunostaining in cancers. Both the frequency of positive cases (blue dots) and the frequency of strongly positive cases (red dots). The conspicuously low rate of strongly positive Whartin tumors is due to the fact, that only basal cells react with p63 resulting in a low overall percentage of positive cells. Fifty five additional tumor entities without any p63 positive cases are not shown due to space restrictions

Nuclear immunostaining was observed in 1940 (19.0%) of 10,200 interpretable tumors with 13.4% showing strong, 3.0% moderate and 2.6% weak staining intensity. At least an occasional weak p63 positivity could be detected in 61 of 115 (53.4%) different tumor types and tumor subtypes and 37 (32.2%) tumor types and tumor subtypes had at least one tumor exhibiting strong positivity. Representative images of p63 positive tumors are shown in Fig. 2. The highest frequencies of p63 positivity were seen in squamous cell carcinomas irrespective of their origin, thymic tumors, urothelial cancers and basal type tumors such as basal cell carcinomas and various salivary gland neoplasia. A detailed description of the immunostaining results is given in Table 1 and Fig. 3.

**Table 1:** p63 immunostaining in human cancers

			p63 immunohistochemistry					
	Tumor type	TMA (n)	analyzable (n)	negative (%)	weak (%)	moderate (%)	strong (%)	positive (%)
**Tumors of the skin**	Pilomatrixoma	35	31	77.4	0.0	6.5	16.1	22.6
	Basal cell carcinoma	48	45	0.0	0.0	0.0	100.0	100.0
	Benign nevus	29	22	100.0	0.0	0.0	0.0	0.0
	Squamous cell carcinoma of the skin	50	47	2.1	2.1	0.0	95.7	97.9
	Malignant melanoma	48	43	97.7	0.0	0.0	2.3	2.3
	Merkel cell carcinoma	46	45	95.6	0.0	4.4	0.0	4.4
**Tumors of the head and neck**	Squamous cell carcinoma of the larynx	100	88	9.1	11.4	15.9	63.6	90.09
	Oral squamous cell cancer	50	47	4.3	0.0	4.3	91.5	95.7
	Oral squamous cell carcinoma (floor of the mouth)	49	48	0.0	6.3	81.3	12.5	100.0
	Pleomorphic adenoma of the parotid gland	15	15	6.7	6.7	6.7	80.0	93.3
	Warthin tumor of the parotid gland	127	83	3.6	4.8	2.4	89.2	96.4
	Basal cell adenoma of the salivary gland	31	16	81.3	18.8	0.0	0.0	18.8
**Tumors of the lung, pleura and thymus**	Adenocarcinoma of the lung	250	189	87.8	10.6	0.0	1.6	12.2
	Squamous cell carcinoma of the lung	6	5	100.0	0.0	0.0	0.0	0.0
	Small cell carcinoma of the lung	20	15	80.0	6.7	6.7	6.7	20.0
	Malignant mesothelioma	39	39	100.0	0.0	0.0	0.0	0.0
	Mesothelioma, other types	76	67	98.5	0.0	1.5	0.0	1.5
	Thymoma	29	29	0.0	13.8	20.7	65.5	100.0
**Tumors of the female genital tract**	Squamous cell carcinoma of the vagina	48	35	2.9	2.9	8.6	85.7	97.1
	Squamous cell carcinoma of the vulva	50	41	0.0	0.0	7.3	92.7	100.0
	Squamous cell carcinoma of the cervix	50	43	2.3	2.3	2.3	93.0	97.7
	Adenocarcinoma of the cervix uteri	50	45	80.0	15.6	4.4	0.0	20.0
	Endometrioid endometrial carcinoma	236	206	83.5	6.8	9.7	0.0	16.5
	Endometrial serous carcinoma	82	60	80.0	11.7	8.3	0.0	20.0
	Endometrial malignant mixed Müllerian tumors (MMMT)	48	40	85.0	7.5	5.0	2.5	15.0
	Endometrial carcinoma, high grade G3	13	12	66.7	0.0	25.0	8.3	33.3
	Endometrial clear cell carcinoma	8	4	100.0	0.0	0.0	0.0	0.0
	Endometrial stromal sarcoma	12	12	100.0	0.0	0.0	0.0	0.0
	Endometrioid carcinoma of the ovary	115	102	87.3	3.9	8.8	0.0	12.7
	Serous carcinoma of the ovary	567	496	92.9	4.2	2.0	0.8	7.1
	Mucinous carcinoma of the ovary	97	81	95.1	2.5	2.5	0.0	4.9
	Clear cell carcinoma of the ovary	54	48	100.0	0.0	0.0	0.0	0.0
	Ovarian malignant mixed Müllerian tumors (MMMT)	47	42	71.4	9.5	19.0	0.0	28.6
	Brenner tumor	9	8	25.0	0.0	0.0	75.0	75.0
**Tumors of the breast**	Invasive breast carcinoma of no special type	126	67	97.0	1.5	1.5	0.0	3.0
	Lobular carcinoma of the breast	123	97	100.0	0.0	0.0	0.0	0.0
	Medullary carcinoma of the breast	15	14	85.7	7.1	7.1	0.0	14.3
	Tubular carcinoma of the breast	18	15	100.0	0.0	0.0	0.0	0.0
	Mucinous carcinoma of the breast	22	16	100.0	0.0	0.0	0.0	0.0
	Phyllodes tumor of the breast	50	36	100.0	0.0	0.0	0.0	0.0
**Tumors of the digestive system**	Adenomatous polyp, low-grade dysplasia	50	41	100.0	0.0	0.0	0.0	0.0
	Adenomatous polyp, high-grade dysplasia	50	43	100.0	0.0	0.0	0.0	0.0
	Adenocarcinoma of the colon	1882	1743	99.6	0.3	0.1	0.0	0.4
	Adenocarcinoma of the small intestine	10	5	100.0	0.0	0.0	0.0	0.0
	Gastric adenocarcinoma, diffuse type	176	153	97.4	1.3	0.0	1.3	2.6
	Gastric adenocarcinoma, intestinal type	174	151	96.0	0.7	0.7	2.6	4.0
	Gastric adenocarcinoma, mixed type	62	56	94.6	1.8	0.0	3.6	5.4
	Adenocarcinoma of the esophagus	83	75	97.3	0.0	0.0	2.7	2.7
	Squamous cell carcinoma of the esophagus	75	63	3.2	1.6	3.2	92.1	96.8
	Squamous cell carcinoma of the anal canal	50	35	0.0	0.0	0.0	100.0	100.0
	Cholangiocarcinoma	50	38	86.8	5.3	0.0	7.9	13.2
	Hepatocellular carcinoma	50	46	100.0	0.0	0.0	0.0	0.0
	Ductal adenocarcinoma of the pancreas	612	489	84.7	5.3	3.7	6.3	15.3
	Pancreatic/Ampullary adenocarcinoma	89	64	92.2	1.6	6.3	0.0	7.8
	Acinar cell carcinoma of the pancreas	13	12	100.0	0.0	0.0	0.0	0.0
	Gastrointestinal stromal tumor (GIST)	50	42	100.0	0.0	0.0	0.0	0.0
**Tumors of the urinary system**	Non-invasive papillary urothelial carcinoma, pTa G2 low grade	177	116	0.0	0.0	2.6	97.4	100.0
	Non-invasive papillary urothelial carcinoma, pTa G2 high grade	141	106	0.0	0.9	8.5	90.6	100.0
	Non-invasive papillary urothelial carcinoma, pTa G3	187	132	9.8	6.1	15.2	68.9	90.2
	Urothelial carcinoma, pT2-4 G3	1117	732	18.9	7.4	9.0	64.8	81.1
	Small cell neuroendocrine carcinoma of the bladder	18	18	88.9	5.6	5.6	0.0	11.1
	Sarcomatoid urothelial carcinoma	25	18	44.4	11.1	5.6	38.9	55.6
	Clear cell renal cell carcinoma	858	509	100.0	0.0	0.0	0.0	0.0
	Papillary renal cell carcinoma	255	155	100.0	0.0	0.0	0.0	0.0
	Clear cell (tubulo) papillary renal cell carcinoma	21	10	100.0	0.0	0.0	0.0	0.0
	Chromophobe renal cell carcinoma	131	96	100.0	0.0	0.0	0.0	0.0
	Oncocytoma	177	110	100.0	0.0	0.0	0.0	0.0
**Tumors of the male genital organs**	Adenocarcinoma of the prostate, Gleason 3+3	83	78	100.0	0.0	0.0	0.0	0.0
	Adenocarcinoma of the prostate, Gleason 4+4	80	71	100.0	0.0	0.0	0.0	0.0
	Adenocarcinoma of the prostate, Gleason 5+5	85	76	100.0	0.0	0.0	0.0	0.0
	Adenocarcinoma of the prostate (recurrence)	330	181	100.0	0.0	0.0	0.0	0.0
	Small cell neuroendocrine carcinoma of the prostate	17	15	100.0	0.0	0.0	0.0	0.0
	Seminoma	50	46	100.0	0.0	0.0	0.0	0.0
	Embryonal carcinoma of the testis	50	42	97.6	0.0	2.4	0.0	2.4
	Yolk sack tumor	50	38	97.4	0.0	2.6	0.0	2.6
	Teratoma	50	33	36.4	9.1	39.4	15.2	63.6
**Tumors of endocrine organs**	Adenoma of the thyroid gland	114	105	100.0	0.0	0.0	0.0	0.0
	Papillary thyroid carcinoma	392	363	89.0	6.1	5.0	0.0	11.0
	Follicular thyroid carcinoma	158	150	98.7	1.3	0.0	0.0	1.3
	Medullary thyroid carcinoma	107	92	100.0	0.0	0.0	0.0	0.0
	Anaplastic thyroid carcinoma	45	38	78.9	2.6	2.6	15.8	21.1
	Adrenal cortical adenoma	50	47	100.0	0.0	0.0	0.0	0.0
	Adrenal cortical carcinoma	26	23	100.0	0.0	0.0	0.0	0.0
	Phaeochromocytoma	50	44	100.0	0.0	0.0	0.0	0.0
	Appendix, neuroendocrine tumor (NET)	22	10	100.0	0.0	0.0	0.0	0.0
	Colorectal, neuroendocrine tumor (NET)	10	7	100.0	0.0	0.0	0.0	0.0
	Ileum, neuroendocrine tumor (NET)	49	41	100.0	0.0	0.0	0.0	0.0
	Lung, neuroendocrine tumor (NET)	19	12	100.0	0.0	0.0	0.0	0.0
	Pancreas, neuroendocrine tumor (NET)	100	83	98.8	1.2	0.0	0.0	1.2
	Colorectal, neuroendocrine carcinoma (NEC)	11	7	85.7	14.3	0.0	0.0	14.3
	Gallbladder, neuroendocrine carcinoma (NEC)	4	2	100.0	0.0	0.0	0.0	0.0
	Pancreas, neuroendocrine carcinoma (NEC)	13	10	100.0	0.0	0.0	0.0	0.0
**Tumors of haemotoppitic and lymphoid tissues**	Hodgkin Lymphoma	45	38	100.0	0.0	0.0	0.0	0.0
	Non-Hodgkin Lymphoma	48	45	60.0	22.2	11.1	6.7	40.0
**Tumors of soft tissue and bone**	Tenosynovial giant cell tumor	45	45	100.0	0.0	0.0	0.0	0.0
	Granular cell tumor	53	50	100.0	0.0	0.0	0.0	0.0
	Leiomyoma	50	47	100.0	0.0	0.0	0.0	0.0
	Angiomyolipoma	91	91	100.0	0.0	0.0	0.0	0.0
	Angiosarcoma	73	66	97.0	0.0	0.0	3.0	3.0
	Dermatofibrosarcoma protuberans	21	21	100.0	0.0	0.0	0.0	0.0
	Ganglioneuroma	14	14	100.0	0.0	0.0	0.0	0.0
	Kaposi sarcoma	8	8	100.0	0.0	0.0	0.0	0.0
	Leiomyosarcoma	87	84	100.0	0.0	0.0	0.0	0.0
	Liposarcoma	132	124	100.0	0.0	0.0	0.0	0.0
	Malignant peripheral nerve sheath tumor (MPNST)	13	13	100.0	0.0	0.0	0.0	0.0
	Myofibrosarcoma	26	26	88.5	11.5	0.0	0.0	11.5
	Neurofibroma	117	117	100.0	0.0	0.0	0.0	0.0
	Sarcoma, not otherwise specified (NOS)	75	75	92.0	8.0	0.0	0.0	8.0
	Paraganglioma	41	41	100.0	0.0	0.0	0.0	0.0
	Primitive neuroectodermal tumor (PNET)	23	23	95.7	0.0	0.0	4.3	4.3
	Rhabdomyosarcoma	7	7	100.0	0.0	0.0	0.0	0.0
	Schwannoma	121	121	100.0	0.0	0.0	0.0	0.0
	Synovial sarcoma	12	12	100.0	0.0	0.0	0.0	0.0
	Osteosarcoma	43	39	94.9	5.1	0.0	0.0	5.1
	Chondrosarcoma	39	27	96.3	3.7	0.0	0.0	3.7

### p63 expression, tumor phenotype and prognosis

**Fig. 4:**
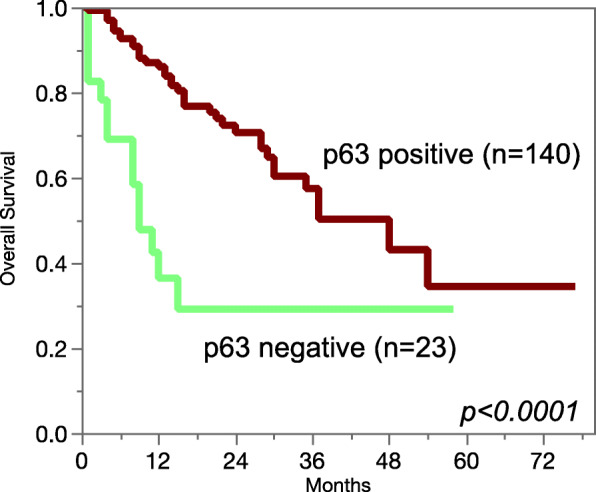
p63 immunostaining and overall survival in urothelial carcinomas. All patients had at least pT2 cancers and were treated by cystectomy

p63 immunostaining was not associated with parameters of disease aggressiveness in 422 pancreatic carcinomas, 160 endometrium cancers, and 374 ovarian cancers (Table 2). In a cohort of 355 gastric carcinomas, p63 positivity was seen in 4% and was linked to nodal metastasis (*p* = 0.0208; Table 2). In urinary bladder cancer, reduced or absent p63 immunostaining was related to advanced stage and high-grade categories (*p* < 0.0001, Table 2) and reduced survival (*p* < 0.0001; Fig. 4).

**Table 2:** p63 immunostaining and tumor phenotype

		n	p63 IHC result (%)				p
			negative	weak	moderate	strong	
Urinary bladder cancer	all cancers	1038	13.4	5.9	9.1	71.7	
	pTa G2 low	116	0.0	0.0	2.6	97.4	<0.0001
	pTa G2 high	106	0.0	0.9	8.5	90.6	
	pTaG3	132	9.8	6.1	15.2	68.9	
	pT≥2 G3	672	18.6	7.0	9.2	65.2	
	pT≥2 G3 sarcomatoid	18	44.4	11.1	5.6	38.9	0.0052 (vs >pT2G3)
	pT≥2 G3 small cell ca.	18	88.9	5.6	5.6	0.0	<0.0001 (vs ≥pT2G3)
	pN0	111	13.5	8.1	9.0	69.4	0.9138
	pN+	71	15.5	5.6	8.5	70.4	
Pancreatic adenocarcinoma	all cancers	422	84.4	5.7	3.1	6.9	
	pT1	12	91.7	0	0	8.3	0.2753
	pT2	63	92.1	3.2	1.6	3.2	
	pT3	318	82.4	6.9	3.1	7.5	
	pT4	27	85.2	0	7.4	7.4	
	G1	12	100	0	0	0	0.3483
	G2	295	82.7	7.1	3.1	7.1	
	G3	92	85.9	3.3	2.2	8.7	
	pN0	91	85.7	3.3	4.4	6.6	0.5777
	pN+	329	83.9	6.4	2.7	7	
	pM0	334	83.2	6.3	3	7.5	0.515
	pM1	87	88.5	3.4	3.4	4.6	
	R0	212	83.5	7.1	2.4	7.1	0.5384
	R1	174	85.6	4.6	4	5.7	
Endometrial cancer, endometrioid	all cancers	160	83.1	4.4	12.5	0.0	
	pT1	101	83.2	3.0	13.9	0.0	0.6733
	pT2	23	87.0	4.3	8.7	0.0	
	pT3-4	33	78.8	9.1	12.1	0.0	
	pN0	43	83.7	4.7	11.6	0.0	0.3704
	pN+	28	75.0	14.3	10.7	0.0	
Ovarian cancers, serous	all cancers	374	91.7	5.1	2.4	0.8	
	pT1	29	93.1	3.4	3.4	0.0	0.2274
	pT2	39	84.6	5.1	10.3	0.0	
	pT3	257	91.8	5.4	1.6	1.2	
	pN0	81	88.9	4.9	4.9	1.2	0.3673
	pN+	166	91.0	6.6	1.2	1.2	
Stomach cancer	all cancers	355	96.1	1.1	0.6	2.3	
	diffuse	85	97.6	2.4	0.0	0.0	0.1478
	intestinal	79	97.5	0.0	0.0	2.5	
	mixed	56	94.6	1.8	0.0	3.6	
	pT1-2	57	96.5	0.0	1.8	1.8	0.3119
	pT3	117	94.9	0.9	0.9	3.4	
	pT4	118	96.6	2.5	0.0	0.8	
	pN0	74	100.0	0.0	0.0	0.0	0.0208
	pN1	62	93.5	1.6	3.2	1.6	
	pN2	55	92.7	0.0	0.0	7.3	
	pN3	101	96.0	3.0	0.0	1.0

## Discussion

The results of our study on 12,620 tissues show that p63 expression is largely limited to few normal tissues including squamous epithelium, urothelium, thymic epithelial cells and basal/myoepithelial cells of various epithelial organs. The fact that p63 expression in these cells is usually strong but completely undetectable in other tissues fits well with the known role of p63 in driving embryonal cellular evolution towards specific cell types. The S-shaped curve displaying the ranking order of p63 positive tumors demonstrates, that frequent and intensive p63 immunostaining is predominantly seen in these few cancers that appear to be derived from p63 positive normal cell types. The most commonly positive cancers include squamous cell carcinomas irrespective of their origin, thymic tumors, urothelial carcinomas and basal type tumors such as basal cell carcinomas and various salivary gland neoplasias.

However, in the majority of p63 positive tumor types, p63 expression was not seen in all cases. Absence of detectable p63 immunostaining in a tumor derived from a p63 expressing normal tissues may in some instances reflect inefficient immunostaining in cancers with inappropriate fixation or other preanalytical issues leading to tissue damage [80]. Our findings in 1038 analyzed urothelial cancers further indicate that p63 expression loss can occur as a result of cellular dedifferentiation during cancer progression. The progressive loss of p63 expression from pTa G2 low grade (0%) small cell urothelial cancer (89%) represents an example of progression associated p63 loss. The fact that p63 loss represents an ominous feature in urothelial carcinomas is also demonstrated by the worse prognosis in p63 negative as compared to p63 positive pT2–4 urothelial carcinomas. These data are in line with reports from other investigators also describing associations of p63 loss with advanced stage and poor prognosis in bladder cancer [41]. Other authors have also described a link between low p63 expression and poor prognosis in squamous cell carcinomas of the esophagus [47] and the larynx [48].

Only nine of 115 cancer types (7.8% of analyzed tumor categories) had a prevalence of p63 positivity between 25 and 90%. These included teratoma and Non-Hodgkin lymphoma. In teratoma the “p63 immunostaining result” of a TMA spot was obviously driven by whether or not epithelial components represented in the spot physiologically expressed p63. In malignant lymphoma, the staining intensity was often moderate. This is reflective of the role of p63 in normal lymphocytes where few cells regularly showed weak to moderate expression. Occasional p63 expression in B-lymphocytes, mainly in germinal centers have been described in previous studies [2, 60]. The high rate of p63 positive B-cell Non-Hodgkin lymphoma is in line with reports from other authors describing common p63 expression, mainly in large cell Non-Hodgkin lymphomas but also in chronic lymphocytic leukemia (CLL), and follicular lymphoma [2, 60, 81–84]. Due to the fact that both the TP63 gene and the Bcl-6 gene are located on chromosome 3q27(− 29) and Bcl-6 gene rearrangements are often seen in diffuse large B-cell lymphomas (DLBCL) [85–88], it has been speculated that the close vicinity of TP63 to Bcl-6 may contribute to its potential involvement in DLBCL tumor progression [60]. However, associations between p63 and BCl-6 expression was not found in another study [84]. Moreover, it remains unclear whether the common translocation at 3q27 affects the expression or structure of the more distal p63 gene [60, 87].

A total of 29 (25%) cancer types and subtypes showed p63 positivity in 3 to 25% of analyzed cases. Very often, p63 staining did not involve the entire tumor mass in these cancers. Almost all of these entities are derived from tissues not normally expressing p63 suggesting neo-expression of p63 occurring during cancer development and progression. At least in a fraction of these tumors p63 neo-expression was obviously linked to focal squamous cell differentiation. Tumor types that are particularly known for occasionally containing squamous elements were common in the category of tumors with p63 positivity between 10 and 25% and included endometroid cancer and malignant mixed Mullerian tumors of the uterus as well as ovarian, pancreatic and cholangiocellular carcinomas. In other tumors with occasional occurrence of p63 positive cells, the phenomenon may reflect stemness properties as earlier shown for p63 expressing normal and cancerous cells [89–91] or be caused by incidental and possibly biologically irrelevant p63 neo-expression in dysregulated cancer cells. The fact that p63 neo-expression was unrelated to features of cancer aggressiveness in cohorts of 422 pancreatic, 374 ovarian, and 160 endometrium cancers argues against a major biologic impact of p63 expression in these tumors. The significantly higher rate of nodal metastasis in p63 positive gastric cancers may be explained by the known poor prognosis of adenosquamous gastric cancers [92].

Overall, these data demonstrate a broad diagnostic utility of p63 IHC for the categorization of cancers, all of which have previously been suggested. For example, p63 expression in a kidney tumor argues for urothelial carcinomas and against poorly differentiated or sarcomatoid renal cell carcinoma in kidney masses [93]. Although several sarcomas showed limited p63 expression, the striking positivity in many sarcomatoid urothelial carcinomas suggests that p63 positivity - in organs where p63 positive epithelial cells are common - argues for sarcomatoid carcinoma and against sarcoma [93, 94]. p63 positivity in a poorly differentiated urinary bladder tumor is literally ruling out infiltration by a prostate cancer even though – considering the general likelihood of these conditions – most p63 negative cancers in the bladder are still representing urothelial carcinomas. In addition, p63 IHC is well established for facilitating the sometimes difficult and clinically highly relevant distinction of adenocarcinoma and squamous cell carcinoma of the lung [22, 52] as well as ruling out invasive cancer by demonstrating a basal cell or myoepithelial cell layer in prostate, breast and salivary gland tumors [24, 67, 95]. It is of note, that in case of a p63 positive solid tumor, Non-Hodgkin lymphoma always remains a diagnostic option.

**Fig. 5:**
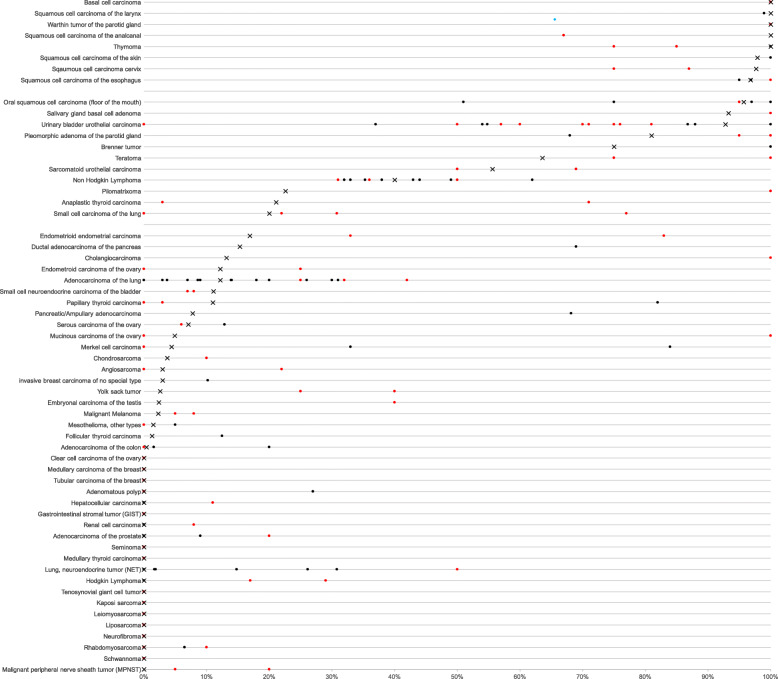
Graphical representation of p63 data from this study (x) in comparison with the previous literature. Red dots are used for studies involving < 25 cases, black dots are used for studies ≥25 cases. All studies are quoted in the list of references

Importantly, all prevalences described in this study are specific to the reagents and the protocol used in our laboratory. The sum of data that had been earlier collected on p63 expression in cancers - summarized in Fig. 5 - would not necessarily have led to the same conclusions as drawn from this study. The use of different antibodies, protocols and interpretation criteria have jointly caused highly diverse literature data on p63 expression in cancer. It is well known, that different antibodies designed for the same target protein can vary to a large extent in their binding properties and that protocol modifications greatly impact the rate of cases considered “positive” for a certain protein [19, 96–102]. A further limitation of the study could be the missing evaluability of about 20% of the tumor samples. However, a statistical bias, which could potentially result from exclusion of non-interpretable samples, is highly unlikely in our study as non-interpretable samples were evenly distributed across all pathological diagnoses.

## Conclusion

Strong and abundant p63 expression is seen in only few cancer types primarily including squamous and urothelial cancers as well as tumors derived from the thymus. Occasional strong p63 expression can, however, occur in a large number of other neoplasias including cancer subtypes with a propensity for focal squamous differentiation. The study also demonstrates the high value of extensive tissue validation using large-scale TMAs for determining the diagnostic utility of antibodies.

## Supplementary Information

**Additional file 1.**

## Data Availability

The datasets used and/or analyzed during the current study are available from the corresponding author on reasonable request.

## Abbreviations

DLBCL: Diffuse Large B Cell Lymphoma
IHC: Immunohistochemistry
TMA: Tissue Micro Array
p63: Tumor Protein 63

## References

[1] Fisher ML, Balinth S, Mills AA. p63-related signaling at a glance. J Cell Sci. 2020;133(17):jcs228015. 10.1242/jcs.228015 PMC7502597 32917730

[2] Di Como CJ, Urist MJ, Babayan I, Drobnjak M, Hedvat CV, Teruya-Feldstein J (2002). p63 expression profiles in human normal and tumor tissues. Clin Cancer Res.

[3] Gatti V, Fierro C, Annicchiarico-Petruzzelli M, Melino G, Peschiaroli A (2019). DeltaNp63 in squamous cell carcinoma: defining the oncogenic routes affecting epigenetic landscape and tumour microenvironment. Mol Oncol.

[4] Murray-Zmijewski F, Lane DP, Bourdon JC (2006). p53/p63/p73 isoforms: an orchestra of isoforms to harmonise cell differentiation and response to stress. Cell Death Differ.

[5] Melino G, Lu X, Gasco M, Crook T, Knight RA (2003). Functional regulation of p73 and p63: development and cancer. Trends Biochem Sci.

[6] Vanbokhoven H, Melino G, Candi E, Declercq W (2011). p63, a story of mice and men. J Invest Dermatol.

[7] Poli Neto OB, Candido dos Reis FJ, Zambelli Ramalho LN, Nogueira AA, de Andrade JM (2006). p63 expression in epithelial ovarian tumors. Int J Gynecol Cancer.

[8] Kaufmann O, Fietze E, Mengs J, Dietel M (2001). Value of p63 and cytokeratin 5/6 as immunohistochemical markers for the differential diagnosis of poorly differentiated and undifferentiated carcinomas. Am J Clin Pathol.

[9] Reis-Filho JS, Simpson PT, Fulford LG, Martins A, Schmitt FC (2003). P63-driven nuclear accumulation of beta-catenin is not a frequent event in human neoplasms. Pathol Res Pract.

[10] Mastropasqua MG, Maiorano E, Pruneri G, Orvieto E, Mazzarol G, Vento AR (2005). Immunoreactivity for c-kit and p63 as an adjunct in the diagnosis of adenoid cystic carcinoma of the breast. Mod Pathol.

[11] Ito Y, Takeda T, Wakasa K, Tsujimoto M, Sakon M, Matsuura N (2001). Expression of p73 and p63 proteins in pancreatic adenocarcinoma: p73 overexpression is inversely correlated with biological aggressiveness. Int J Mol Med.

[12] Ramalho FS, Ramalho LN, Della Porta L, Zucoloto S (2006). Comparative immunohistochemical expression of p63 in human cholangiocarcinoma and hepatocellular carcinoma. J Gastroenterol Hepatol.

[13] Dickson BC, Li SQ, Wunder JS, Ferguson PC, Eslami B, Werier JA (2008). Giant cell tumor of bone express p63. Mod Pathol.

[14] Gualco G, Weiss LM, Bacchi CE (2008). Expression of p63 in anaplastic large cell lymphoma but not in classical Hodgkin’s lymphoma. Hum Pathol.

[15] Bir F, Aksoy Altinboga A, Satiroglu Tufan NL, Kaya S, Baser S, Yaren A (2014). Potential utility of p63 expression in differential diagnosis of non-small-cell lung carcinoma and its effect on prognosis of the disease. Med Sci Monit.

[16] Zhang C, Schmidt LA, Hatanaka K, Thomas D, Lagstein A, Myers JL (2014). Evaluation of napsin A, TTF-1, p63, p40, and CK5/6 immunohistochemical stains in pulmonary neuroendocrine tumors. Am J Clin Pathol.

[17] Righi L, Graziano P, Fornari A, Rossi G, Barbareschi M, Cavazza A (2011). Immunohistochemical subtyping of nonsmall cell lung cancer not otherwise specified in fine-needle aspiration cytology: a retrospective study of 103 cases with surgical correlation. Cancer..

[18] Stojsic J, Jovanic I, Markovic J, Gajic M (2013). Contribution of immunohistochemistry in the differential diagnosis of non-small cell lung carcinomas on small biopsy samples. J Buon.

[19] Au NH, Gown AM, Cheang M, Huntsman D, Yorida E, Elliott WM (2004). P63 expression in lung carcinoma: a tissue microarray study of 408 cases. Appl Immunohistochem Mol Morphol.

[20] Yamada K, Maeshima AM, Tsuta K, Tsuda H (2014). Combined high-grade neuroendocrine carcinoma of the lung: clinicopathological and immunohistochemical study of 34 surgically resected cases. Pathol Int.

[21] Wu M, Wang B, Gil J, Sabo E, Miller L, Gan L (2003). p63 and TTF-1 immunostaining. A useful marker panel for distinguishing small cell carcinoma of lung from poorly differentiated squamous cell carcinoma of lung. Am J Clin Pathol.

[22] Bishop JA, Teruya-Feldstein J, Westra WH, Pelosi G, Travis WD, Rekhtman N (2012). p40 (DeltaNp63) is superior to p63 for the diagnosis of pulmonary squamous cell carcinoma. Mod Pathol.

[23] Baydar DE, Kulac I, Gurel B, De Marzo A (2011). A case of prostatic adenocarcinoma with aberrant p63 expression: presentation with detailed immunohistochemical study and FISH analysis. Int J Surg Pathol.

[24] Bilal H, Handra-Luca A, Bertrand JC, Fouret PJ (2003). P63 is expressed in basal and myoepithelial cells of human normal and tumor salivary gland tissues. J Histochem Cytochem.

[25] Genelhu MC, Gobbi H, Soares FA, Campos AH, Ribeiro CA, Cassali GD (2006). Immunohistochemical expression of p63 in pleomorphic adenomas and carcinomas ex-pleomorphic adenomas of salivary glands. Oral Oncol.

[26] Owosho AA, Aguilar CE, Seethala RR (2016). Comparison of p63 and p40 (DeltaNp63) as basal, squamoid, and myoepithelial markers in salivary gland tumors. Appl Immunohistochem Mol Morphol.

[27] Seethala RR, LiVolsi VA, Zhang PJ, Pasha TL, Baloch ZW (2005). Comparison of p63 and p73 expression in benign and malignant salivary gland lesions. Head Neck.

[28] Daguci L, Stepan A, Mercut V, Daguci C, Bataiosu M, Florescu A (2012). Immunohistochemical expression of CK7, CK5/6, CK19, and p63 in Warthin tumor. Romanian J Morphol Embryol.

[29] Vidal CI, Goldberg M, Burstein DE, Emanuel HJ, Emanuel PO (2010). p63 immunohistochemistry is a useful adjunct in distinguishing sclerosing cutaneous tumors. Am J Dermatopathol.

[30] Kanitakis J, Chouvet B (2007). Expression of p63 in cutaneous metastases. Am J Clin Pathol.

[31] Gleason BC, Calder KB, Cibull TL, Thomas AB, Billings SD, Morgan MB (2009). Utility of p63 in the differential diagnosis of atypical fibroxanthoma and spindle cell squamous cell carcinoma. J Cutan Pathol.

[32] Jo VY, Fletcher CD (2011). p63 immunohistochemical staining is limited in soft tissue tumors. Am J Clin Pathol.

[33] Westfall DE, Folpe AL, Paner GP, Oliva E, Goldstein L, Alsabeh R (2009). Utility of a comprehensive immunohistochemical panel in the differential diagnosis of spindle cell lesions of the urinary bladder. Am J Surg Pathol.

[34] Emanuel PO, Unger PD, Burstein DE (2006). Immunohistochemical detection of p63 in testicular germ cell neoplasia. Ann Diagn Pathol.

[35] Su XY, Wang WY, Li JN, Liao DY, Wu WL, Li GD (2015). Immunohistochemical differentiation between type B3 thymomas and thymic squamous cell carcinomas. Int J Clin Exp Pathol.

[36] Kim YW, Do IG, Park YK (2006). Expression of the GLUT1 glucose transporter, p63 and p53 in thyroid carcinomas. Pathol Res Pract.

[37] Preto A, Reis-Filho JS, Ricardo S, Soares P (2002). P63 expression in papillary and anaplastic carcinomas of the thyroid gland: lack of an oncogenetic role in tumorigenesis and progression. Pathol Res Pract.

[38] Paner GP, Annaiah C, Gulmann C, Rao P, Ro JY, Hansel DE (2014). Immunohistochemical evaluation of novel and traditional markers associated with urothelial differentiation in a spectrum of variants of urothelial carcinoma of the urinary bladder. Hum Pathol.

[39] Rajcani J, Kajo K, Adamkov M, Moravekova E, Lauko L, Felcanova D (2013). Immunohistochemical characterization of urothelial carcinoma. Bratisl Lek Listy.

[40] Buza N, Cohen PJ, Pei H, Parkash V (2010). Inverse p16 and p63 expression in small cell carcinoma and high-grade urothelial cell carcinoma of the urinary bladder. Int J Surg Pathol.

[41] Lin X, Zhu B, Villa C, Zhong M, Kundu S, Rohan SM (2014). The utility of p63, p40, and GATA-binding protein 3 immunohistochemistry in diagnosing micropapillary urothelial carcinoma. Hum Pathol.

[42] Grapsa D, Dokou A, Tsokanou-Kouli V, Kaltsas S, Dalakou E, Trigidou R (2014). Immunohistochemical expression of p53, p63, c-myc, p21(WAF1/cip1) and p27(kip1) proteins in urothelial bladder carcinoma: correlation with clinicopathological parameters. J Buon.

[43] Thompson S, Cioffi-Lavina M, Chapman-Fredricks J, Gomez-Fernandez C, Fernandez-Castro G, Jorda M (2011). Distinction of high-grade neuroendocrine carcinoma/small cell carcinoma from conventional urothelial carcinoma of urinary bladder: an immunohistochemical approach. Appl Immunohistochem Mol Morphol.

[44] Wang X, Boddicker RL, Dasari S, Sidhu JS, Kadin ME, Macon WR (2017). Expression of p63 protein in anaplastic large cell lymphoma: implications for genetic subtyping. Hum Pathol.

[45] Guo HQ, Huang GL, Liu OF, Liu YY, Yao ZH, Yao SN (2012). p63 expression is a prognostic factor in colorectal cancer. Int J Biol Markers.

[46] Hara T, Kijima H, Yamamoto S, Kenmochi T, Kise Y, Tanaka H (2004). Ubiquitous p63 expression in human esophageal squamous cell carcinoma. Int J Mol Med.

[47] Takahashi Y, Noguchi T, Takeno S, Kimura Y, Okubo M, Kawahara K (2006). Reduced expression of p63 has prognostic implications for patients with esophageal squamous cell carcinoma. Oncol Rep.

[48] Borba M, Cernea C, Dias F, Faria P, Bacchi C, Brandao L (2010). Expression profile of p63 in 127 patients with laryngeal squamous cell carcinoma. ORL J Otorhinolaryngol Relat Spec.

[49] Aubry MC, Roden A, Murphy SJ, Vasmatzis G, Johnson SH, Harris FR (2015). Chromosomal rearrangements and copy number abnormalities of TP63 correlate with p63 protein expression in lung adenocarcinoma. Mod Pathol.

[50] Nonaka D (2012). A study of DeltaNp63 expression in lung non-small cell carcinomas. Am J Surg Pathol.

[51] Whithaus K, Fukuoka J, Prihoda TJ, Jagirdar J (2012). Evaluation of napsin A, cytokeratin 5/6, p63, and thyroid transcription factor 1 in adenocarcinoma versus squamous cell carcinoma of the lung. Arch Pathol Lab Med.

[52] Conde E, Angulo B, Redondo P, Toldos O, Garcia-Garcia E, Suarez-Gauthier A (2010). The use of P63 immunohistochemistry for the identification of squamous cell carcinoma of the lung. PLoS One.

[53] Narahashi T, Niki T, Wang T, Goto A, Matsubara D, Funata N (2006). Cytoplasmic localization of p63 is associated with poor patient survival in lung adenocarcinoma. Histopathology..

[54] Xu XY, Yang GY, Yang JH, Li J (2014). Analysis of clinical characteristics and differential diagnosis of the lung biopsy specimens in 99 adenocarcinoma cases and 111 squamous cell carcinoma cases: utility of an immunohistochemical panel containing CK5/6, CK34betaE12, p63, CK7 and TTF-1. Pathol Res Pract.

[55] Montezuma D, Azevedo R, Lopes P, Vieira R, Cunha AL, Henrique R (2013). A panel of four immunohistochemical markers (CK7, CK20, TTF-1, and p63) allows accurate diagnosis of primary and metastatic lung carcinoma on biopsy specimens. Virchows Arch.

[56] Hall BJ, Pincus LB, Yu SS, Oh DH, Wilson AR, McCalmont TH (2012). Immunohistochemical prognostication of Merkel cell carcinoma: p63 expression but not polyomavirus status correlates with outcome. J Cutan Pathol.

[57] Stetsenko GY, Malekirad J, Paulson KG, Iyer JG, Thibodeau RM, Nagase K (2013). p63 expression in Merkel cell carcinoma predicts poorer survival yet may have limited clinical utility. Am J Clin Pathol.

[58] Rakha EA, Coimbra ND, Hodi Z, Juneinah E, Ellis IO, Lee AH (2017). Immunoprofile of metaplastic carcinomas of the breast. Histopathology..

[59] Pruneri G, Fabris S, Dell’Orto P, Biasi MO, Valentini S, Del Curto B (2005). The transactivating isoforms of p63 are overexpressed in high-grade follicular lymphomas independent of the occurrence of p63 gene amplification. J Pathol.

[60] Hedvat CV, Teruya-Feldstein J, Puig P, Capodieci P, Dudas M, Pica N (2005). Expression of p63 in diffuse large B-cell lymphoma. Appl Immunohistochem Mol Morphol.

[61] Xu-Monette ZY, Zhang S, Li X, Manyam GC, Wang XX, Xia Y (2016). p63 expression confers significantly better survival outcomes in high-risk diffuse large B-cell lymphoma and demonstrates p53-like and p53-independent tumor suppressor function. Aging (Albany NY).

[62] Foschini MP, Gaiba A, Cocchi R, Pennesi MG, Gatto MR, Frezza GP (2004). Pattern of p63 expression in squamous cell carcinoma of the oral cavity. Virchows Arch.

[63] Moergel M, Abt E, Stockinger M, Kunkel M (2010). Overexpression of p63 is associated with radiation resistance and prognosis in oral squamous cell carcinoma. Oral Oncol.

[64] Lo Muzio L, Santarelli A, Caltabiano R, Rubini C, Pieramici T, Trevisiol L (2005). p63 overexpression associates with poor prognosis in head and neck squamous cell carcinoma. Hum Pathol.

[65] Saghravanian N, Anvari K, Ghazi N, Memar B, Shahsavari M, Aghaee MA (2017). Expression of p63 and CD44 in oral squamous cell carcinoma and correlation with clinicopathological parameters. Arch Oral Biol.

[66] Ud Din N, Qureshi A, Mansoor S (2011). Utility of p63 immunohistochemical stain in differentiating urothelial carcinomas from adenocarcinomas of prostate. Indian J Pathol Microbiol.

[67] Uchida K, Ross H, Lotan T, Pignon JC, Signoretti S, Epstein JI (2015). DeltaNp63 (p40) expression in prostatic adenocarcinoma with diffuse p63 positivity. Hum Pathol.

[68] Parsons JK, Gage WR, Nelson WG, De Marzo AM (2001). p63 protein expression is rare in prostate adenocarcinoma: implications for cancer diagnosis and carcinogenesis. Urology..

[69] Tuna B, Unlu M, Aslan G, Secil M, Yorukoglu K (2009). Diagnostic and prognostic impact of p63 immunoreactivity in renal malignancies. Anal Quant Cytol Histol.

[70] Langner C, Ratschek M, Tsybrovskyy O, Schips L, Zigeuner R (2003). P63 immunoreactivity distinguishes upper urinary tract transitional-cell carcinoma and renal-cell carcinoma even in poorly differentiated tumors. J Histochem Cytochem.

[71] Rooper L, Sharma R, Bishop JA (2015). Polymorphous low grade adenocarcinoma has a consistent p63+/p40- immunophenotype that helps distinguish it from adenoid cystic carcinoma and cellular pleomorphic adenoma. Head Neck Pathol.

[72] Valencia-Guerrero A, Dresser K, Cornejo KM (2018). Utility of immunohistochemistry in distinguishing primary adnexal carcinoma from metastatic breast carcinoma to skin and squamous cell carcinoma. Am J Dermatopathol.

[73] Dotto J, Pelosi G, Rosai J (2007). Expression of p63 in thymomas and normal thymus. Am J Clin Pathol.

[74] Vrabie CD, Terzea D, Petrescu A, Waller M (2009). The histopathology analysis of the diffuse sclerosing variant of the papillary carcinoma of the thyroid: a distinctive and rare form. Romanian J Morphol Embryol.

[75] Necchi A, Giannatempo P, Paolini B, Lo Vullo S, Marongiu M, Fare E (2015). Immunohistochemistry to enhance prognostic allocation and guide decision-making of patients with advanced urothelial cancer receiving first-line chemotherapy. Clin Genitourin Cancer.

[76] Koyuncuer A (2013). Immunohistochemical expression of p63, p53 in urinary bladder carcinoma. Indian J Pathol Microbiol.

[77] Carneiro FP, Ramalho LN, Britto-Garcia S, Ribeiro-Silva A, Zucoloto S (2006). Immunohistochemical expression of p16, p53, and p63 in colorectal adenomas and adenocarcinomas. Dis Colon Rectum.

[78] Zhang N, Huo Q, Wang X, Chen X, Long L, Guan X (2014). A genetic variant in p63 (rs17506395) is associated with breast cancer susceptibility and prognosis. Gene..

[79] Reis-Filho JS, Simpson PT, Martins A, Preto A, Gartner F, Schmitt FC (2003). Distribution of p63, cytokeratins 5/6 and cytokeratin 14 in 51 normal and 400 neoplastic human tissue samples using TARP-4 multi-tumor tissue microarray. Virchows Arch.

[80] Fraune C, Simon R, Hube-Magg C, Makrypidi-Fraune G, Kahler C, Kluth M (2020). MMR deficiency in urothelial carcinoma of the bladder presents with temporal and spatial homogeneity throughout the tumor mass. Urol Oncol.

[81] Park CK, Oh YH (2005). Expression of p63 in reactive hyperplasias and malignant lymphomas. J Korean Med Sci.

[82] Fukushima N, Satoh T, Sueoka N, Sato A, Ide M, Hisatomi T (2006). Clinico-pathological characteristics of p63 expression in B-cell lymphoma. Cancer Sci.

[83] Robson A, Shukur Z, Ally M, Kluk J, Liu K, Pincus L (2016). Immunocytochemical p63 expression discriminates between primary cutaneous follicle centre cell and diffuse large B cell lymphoma-leg type, and is of the TAp63 isoform. Histopathology..

[84] Hallack Neto AE, Siqueira SA, Dulley FL, Ruiz MA, Chamone DA, Pereira J (2009). p63 protein expression in high risk diffuse large B-cell lymphoma. J Clin Pathol.

[85] Liang R, Chan WP, Kwong YL, Xu WS, Srivastava G, Ho FC (1997). High incidence of BCL-6 gene rearrangement in diffuse large B-cell lymphoma of primary gastric origin. Cancer Genet Cytogenet.

[86] Skinnider BF, Horsman DE, Dupuis B, Gascoyne RD (1999). Bcl-6 and Bcl-2 protein expression in diffuse large B-cell lymphoma and follicular lymphoma: correlation with 3q27 and 18q21 chromosomal abnormalities. Hum Pathol.

[87] Yang A, Kaghad M, Wang Y, Gillett E, Fleming MD, Dotsch V (1998). p63, a p53 homolog at 3q27-29, encodes multiple products with transactivating, death-inducing, and dominant-negative activities. Mol Cell.

[88] Dalla-Favera R, Ye BH, Lo Coco F, Chang CC, Cechova K, Zhang J (1994). BCL-6 and the molecular pathogenesis of B-cell lymphoma. Cold Spring Harb Symp Quant Biol.

[89] Liu Y, Nekulova M, Nenutil R, Horakova I, Appleyard MV, Murray K (2020). Np63/p40 correlates with the location and phenotype of basal/mesenchymal cancer stem-like cells in human ER(+) and HER2(+) breast cancers. J Pathol Clin Res.

[90] Timofeeva OA, Palechor-Ceron N, Li G, Yuan H, Krawczyk E, Zhong X (2017). Conditionally reprogrammed normal and primary tumor prostate epithelial cells: a novel patient-derived cell model for studies of human prostate cancer. Oncotarget..

[91] Pignon JC, Grisanzio C, Geng Y, Song J, Shivdasani RA, Signoretti S (2013). p63-expressing cells are the stem cells of developing prostate, bladder, and colorectal epithelia. Proc Natl Acad Sci U S A.

[92] Akce M, Jiang R, Alese OB, Shaib WL, Wu C, Behera M (2019). Gastric squamous cell carcinoma and gastric adenosquamous carcinoma, clinical features and outcomes of rare clinical entities: a National Cancer Database (NCDB) analysis. J Gastrointest Oncol.

[93] Truong LD, Shen SS (2011). Immunohistochemical diagnosis of renal neoplasms. Arch Pathol Lab Med.

[94] Reis-Filho JS, Schmitt FC (2003). p63 expression in sarcomatoid/metaplastic carcinomas of the breast. Histopathology..

[95] Stefanou D, Batistatou A, Nonni A, Arkoumani E, Agnantis NJ (2004). p63 expression in benign and malignant breast lesions. Histol Histopathol.

[96] Acs G, Acs P, Beckwith SM, Pitts RL, Clements E, Wong K (2001). Erythropoietin and erythropoietin receptor expression in human cancer. Cancer Res.

[97] Andersson S, Sundberg M, Pristovsek N, Ibrahim A, Jonsson P, Katona B (2017). Corrigendum: insufficient antibody validation challenges oestrogen receptor beta research. Nat Commun.

[98] Elliott S, Swift S, Busse L, Scully S, Van G, Rossi J (2013). Epo receptors are not detectable in primary human tumor tissue samples. PLoS One.

[99] Laflamme C, McKeever PM, Kumar R, Schwartz J, Kolahdouzan M, Chen CX (2019). Implementation of an antibody characterization procedure and application to the major ALS/FTD disease gene C9ORF72. Elife..

[100] Sinclair AM, Todd MD, Forsythe K, Knox SJ, Elliott S, Begley CG (2007). Expression and function of erythropoietin receptors in tumors: implications for the use of erythropoiesis-stimulating agents in cancer patients. Cancer..

[101] Trincavelli ML, Da Pozzo E, Ciampi O, Cuboni S, Daniele S, Abbracchio MP (2013). Regulation of erythropoietin receptor activity in endothelial cells by different erythropoietin (EPO) derivatives: an in vitro study. Int J Mol Sci.

[102] Saper CB (2009). A guide to the perplexed on the specificity of antibodies. J Histochem Cytochem.

