# Supplementary material for: p63 expression in human tumors and normal tissues: a tissue microarray study on 10,200 tumors
Source: Biomark Res. 2021 Jan 25;9:7. doi: 10.1186/s40364-021-00260-5 (PMC7830855; doi:10.1186/s40364-021-00260-5)
Supplement: Supplementary file 1 — Additional file 1. [file 40364_2021_260_MOESM1_ESM.docx]

|  |  | n | **p63 IHC interpretable (%)** | |
| --- | --- | --- | --- | --- |
|  |  |  | yes | no |
|  |  |  |  |  |
|  | pTa G2 low | 177 | 65.5 | 34.5 |
|  | pTa G2 high | 141 | 75.2 | 24.8 |
|  | pTaG3 | 187 | 70.6 | 29.4 |
|  | pT≥2 G3 | 890 | 76.9 | 23.2 |
|  |  |  |  |  |
|  | pT≥2 G3 sarcomatoid | 25 | 72.0 | 28.0 |
|  | pT≥2 G3 small cell ca. | 18 | 100.0 | 0.0 |
|  |  |  |  |  |
|  | pN0 | 173 | 64.2 | 35.8 |
|  | pN+ | 95 | 74.7 | 25.3 |
